# Clinical variables responsible for early and late diagnosis of foreign body aspiration in pediatrics age group

**DOI:** 10.1186/s13019-020-01314-9

**Published:** 2020-09-29

**Authors:** Samarth Goyal, Shubhika Jain, Guruprasad Rai, Rajkamal Vishnu, Ganesh Sevagur Kamath, Arvind Kumar Bishnoi, Yogesh Gaude, Vijaya Kumara, Harshil Joshi, Revanth Reddy

**Affiliations:** 1 Kasturba Medical College, Manipal Academy of Higher Education, Manipal, Udupi, Karnataka 576 104 India; 2 Department of Cardiothoracic and vascular surgery, Kasturba Medical College, Manipal Academy of Higher Education, Manipal, Udupi, Karnataka 576 104 India; 3 Department of Anesthesiology, Kasturba Medical College, Manipal Academy of Higher Education, Manipal, Udupi, Karnataka 576 104 India

**Keywords:** Bronchus, Foreign body (FB), Foreign body aspiration (FBA), Rigid bronchoscopy

## Abstract

**Background:**

Incidence of foreign body aspiration has been noticed predominantly in age group ranging from 12 months-3 years. Foreign body in the trachea is a medical emergency as presentation is in respiratory distress. Obstruction of only one main or distal bronchus, leads to severe cough, choking sensation and breathlessness. Without early intervention, it can lead to collapse, consolidation and pneumonia of the affected lung.

**Methods:**

We retrospectively analyzed 37 pediatric case records who presented from January 2014–December 2018 with foreign body aspiration. Our primary aim was to assess the parameters responsible for early and late diagnosis of foreign body aspiration. We concluded with a diagnostic algorithm for management of foreign body aspiration on the basis of this outcome.

**Results:**

Around 32.5% came with a history of aspiration, 43% were referred from the primary centers with a suspicion for the same and the rest came to our tertiary care hospital directly. Those who presented within a week came with complaints of wet cough, wheeze and tachypnea. Furthermore, those who came in after a week had a dry cough and fever as their main complaint. Majority of ingested foreign bodies was a vegetative type (80%) as compared to the non –vegetative.

**Conclusion:**

Unlike adults, foreign body aspiration in children is most commonly diagnosed on history, suspicion and clinical findings. Chest x ray has been the primary investigation of choice but in the majority of the cases it was normal with subtle changes. Early diagnosis is the key to avoid complication.

## Background

Foreign body aspiration is an egregious medical emergency [1]. Both adults and children have a tendency to inhale foreign bodies. The object enters into the trachea and usually goes further down and lodges in one of the bronchus. Children have a tendency to swallow whatever comes into their hands, which include a wide variety of objects like coin, parts of toys, seeds, nuts, etc. and when they cry or laugh or jump with the mouth full, the FB can enter the airway in deep inspiration. In adult’s the main cause for aspiration is trying to swallow food when they are under intoxication and common FB are unchewed food, fish bone, etc. FBA is said to be a primary cause of accidental death in children under 12 months to 3 years of age [2]. Literatures suggest sex discrepancy of 60% with the majority of the patients being males [3]. FB in the airway can lead to choking and ultimately death due to asphyxia if the diagnosis is delayed [4]. National Safety Council of America in 2016 proposed the rate of fatal choking in children < 5 years of age in the American general population was 0.43 per 100,000 [5].

We aimed to analyze retrospectively and determine clinical predictors like, history of presentation, physical and radiological findings which help in early diagnosis. We also intend to study the parameters which lead to delayed diagnosis and draw an algorithm for management on the basis of our experience.

## Methods

We conducted this retrospective study at the Kasturba Medical College Hospital, Manipal between January 2014 and December 2018. Institutional ethical committee clearance was obtained before the initiation of the study. A retrospective analysis of the medical records of 37 consecutive patients of pediatric age group who were treated in our tertiary care hospital for FBA aspiration were included in the study.

We analyzed the following details from the medical records: demographic profile (age and gender), time of onset of symptoms and history of treatment at a local hospital, time of referral to our hospital and the treatment given, the duration from the onset of symptoms and the referral. The clinical features, physical examination and radiological findings at presentation were noted. Bronchoscopy findings were also tabulated. Final result and complications were documented.

Data was entered and analyzed using Statistical Package for Social Sciences (SPSS) version 15. The results are summarized as percentages and proportions.

## Results

Total of 37 case files were analysed. The socio-demographic data of the patients is depicted in Table 1.

**Table 1:** Socio-Demographic details

	*n* = 37	%
**Age**		
1–3 years	32	86.4
> 3 years	5	13.6
**Gender**		
Male	25	67.5
Female	12	32.5

Out of 37 cases studied, 12 (32.4%) came with a history of foreign body aspiration. Sixteen cases (43.2%) were referred to the hospital from the primary centers with suspicion of foreign body whereas remaining presented to the hospital directly.

**Fig. 1:**
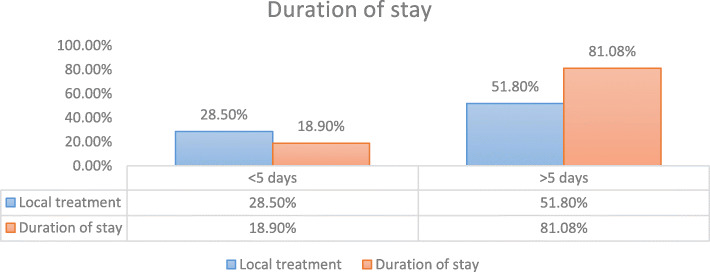
Relation of duration of stay in the hospital and local treatment

Delay in presentation was more in patient who were initially treated symptomatically in a primary health center is outlined in Table 2. Of 20 patients who came to our hospital after 1 week, 13 (65%) were treated locally, whereas who presented before 1 week of onset of symptoms only 3 (17.7%) out of 17 underwent previous treatment (Fig. 1).

**Table 2:** Time duration between onset of symptom and presentation to hospital

	***n*** = 37	%
Within 24 h.	9	24.32
1 day to 1 week	8	21.6%
1 week to 1 month	10	27.02
> 1 month	10	27.02

**Fig. 2:**
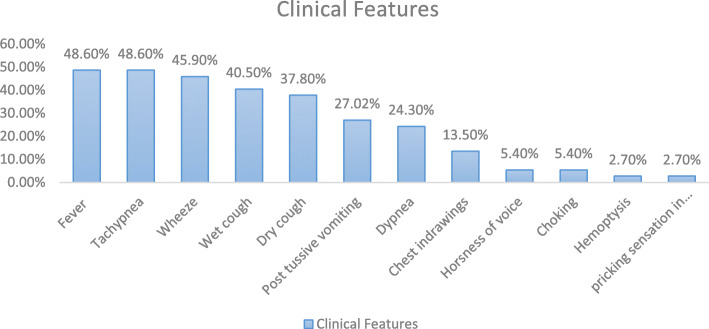
Clinical manifestation at time of presentation

Most common presenting features in cases that presented early (< 1 week) were Tachypnea (58.8%), Wheeze (47.05%) and wet cough (41.1%). Cases that presented late (> 1 week) came mainly with complain of fever (65%) and dry cough (45%) (Fig. 2). History of foreign body aspiration was present in 47.05% of early cases and 20% of late cases.

**Fig. 3:**
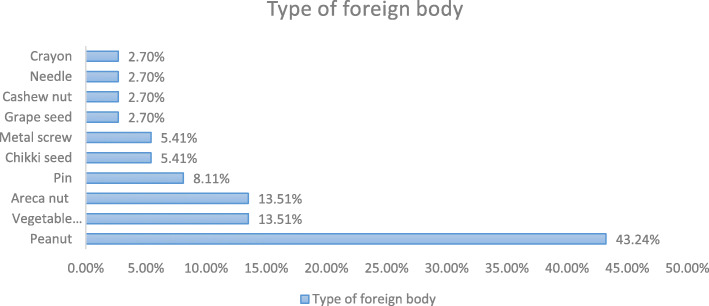
Types of foreign body

Eighty percent of foreign bodies were of vegetative types, peanut being most common (43.24%) and 18.9% included non-vegetative material like metal screw, pin, needle and crayon (Fig. 3).

The location of foreign body and related examination and radiological findings are discussed in Table 3.

**Table 3:** Location of foreign body and related examination and radiological findings

Location on the basis of bronchoscopy	Respiratory examination		Radiological findings (x ray)	
Left bronchus			X ray done	100%
	Decreased left sided chest movements	26.3%		
			X ray showed left sided FB	42.1%
	Decreased air entry on left side	47.4%	Left lung collapse	21.1%
	Decreased air entry on right side	5.3%	Mediastinal shift to left	5.3%
	Rhonchi	36.9%		
			Mediastinal shift to right	5.3%
	Crepitus	31.6%		
Right bronchus			X ray done	100%
	Decreased right sided chest movements	5.9%	X ray showed right sided FB	41.4%
			Right lung collapse	17.7%
	Decreased air entry on right side	64.7%	Mediastinal shift to right	5.9%
	Decreased air entry on left side	5.9%	Mediastinal shift to left	11.8%
	Rhonchi	47.1%	Pericardial pneumonitis	11.8%
	Crepitus	17.7%		

All the cases underwent rigid bronchoscopy once. In 4 cases we had to do flexible bronchoscopy for inspection due to; incomplete removal, slippage of some part into a segmental bronchus and impaction of FB to the mucosa.

Out of 37 cases, 35 cases (94.6%) have undergone rigid bronchoscopic removal. Adjuvant treatment that was given along with bronchoscopic removal was antibiotics (72.97%), bronchodilators (29.73%) and steroids (18.90%). In 2 cases (5.4%) required surgical retrieval.

## Discussion

Foreign-body aspiration accounts for high morbidity if the diagnosis is delayed or missed. Mortality also is reported in children, especially between ages 12 months to 3 years [2]. Most children under the age of 3 years tend to mull over most particles in their mouths, they also have flawed nibbling habits and premature swallowing coordination which makes them more prone for a FBA. Children are more susceptible to some FBA complications due to immature defense mechanisms [6]. Often the presentation and initial radiological findings are vague, which impedes the early diagnosis. An alleged episode of choking and severe cough is a critical evidence in the diagnosis of FB aspiration.

In our study, choking (5.40%) and hemoptysis (2.70%) were seen in limited cases. The most common symptoms in patients who presented early (< 1 week) were tachypnea (58.8%), wheeze (47.05%) and wet cough (41.1%). In cases of delayed presentation (> 1 week) majority complained of fever (65%) and dry cough (45%). It was found that the delay in presentation was mostly due to unintentional aspiration by child unnoticed by parents, vague clinical history, lack of respiratory symptoms, unwillingness by the treating physician for getting a chest X ray and delayed referral. It was noted that the duration of treatment at the local hospital was directly proportional to the duration of admission later for effective management.

**Fig. 4:**
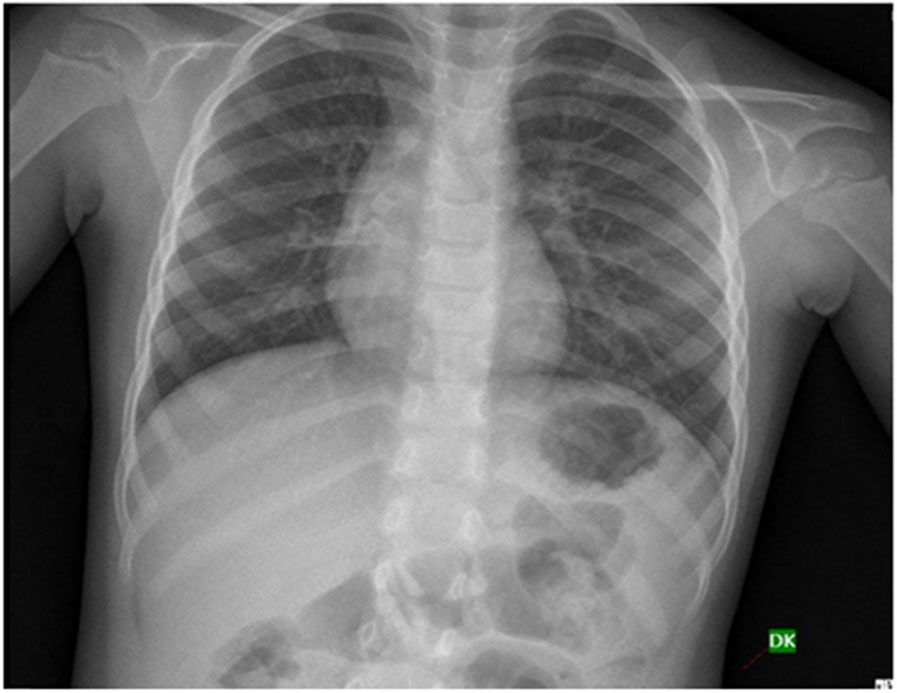
Normal Chest x-ray in a case of early FBA

Unlike adults, toddlers do not have clear recall of FBA, thus the diagnosis is most often dependent on the mother’s history, clinical findings such as onset and duration of symptoms and suspicion. Chest x -ray is the most common investigation done in these cases and majority of the time chest- x rays are normal (Fig. 4).

The typical findings on chest radiograph which is diagnostic is unilateral lung hyperinflation, collapse, consolidation of one lung and mediastinal shift. Many authors in their study have reported percentage of normal chest X -ray around 20–42% [7, 8]. In our study normal chest x-ray was found in 39% of patients. Normal chest X-ray is accepted in early phase as most aspirated foreign bodies are vegetative in nature and cannot be seen on chest x ray.

**Fig. 5:**
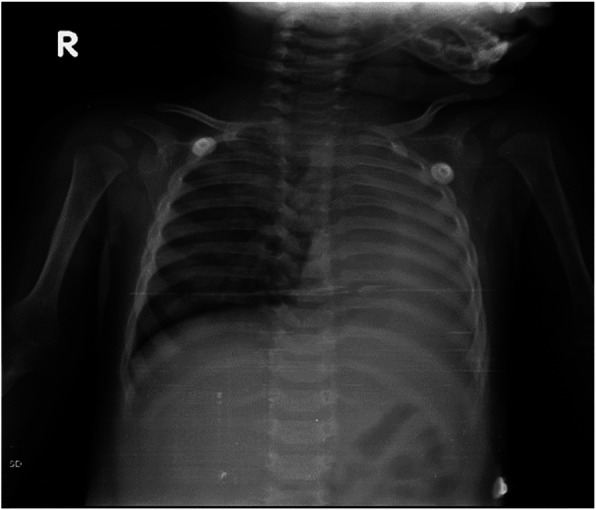
Chest x-ray showing collapse, consolidation and mediastinal shift of left lung due to long standing vegetative FB

Later, when the organic substance swells with inflammation it occludes the bronchus completely and typical chest x-ray findings appear (Fig. 5).

**Fig. 6:**
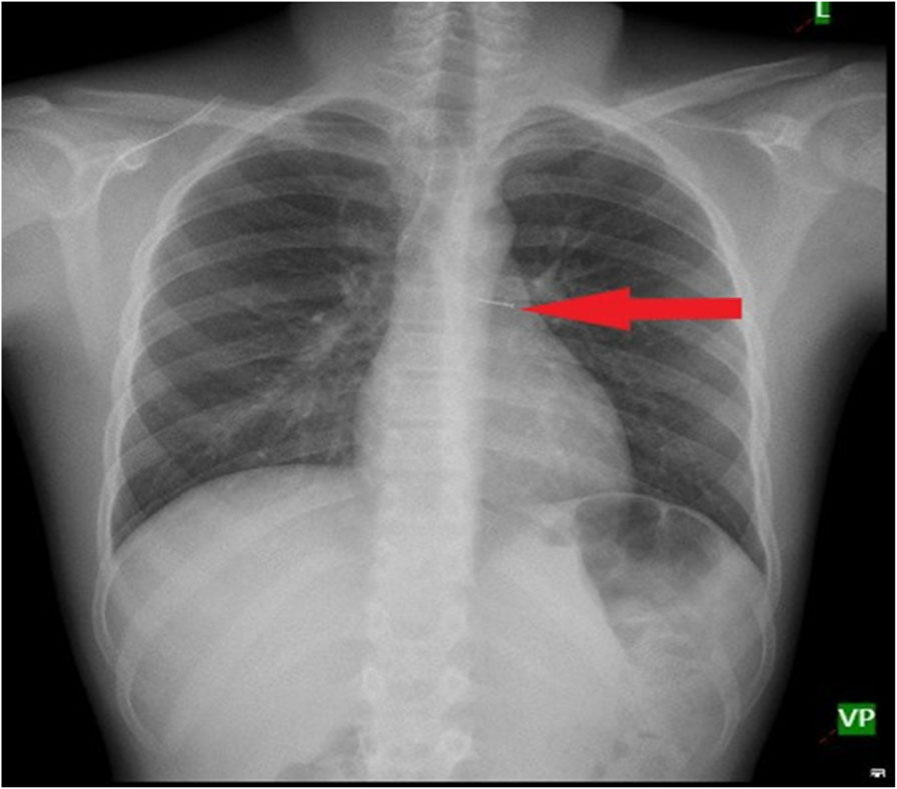
Chest x-ray showing pin in the left main bronchus

On the contrary, most of the metallic objects are relatively radiolucent and easily picked up on chest X ray and aids clinicians in early diagnosis of FBA (Fig. 6).

Recently Computerized tomography (CT) has been introduced as a noninvasive procedure in the diagnosis of FBA, but it is known to show false positive results and also has radiation hazards associated with it [9–11].

Various other diagnostic modalities have been reported to be effective, rigid bronchoscopic inspection being the gold standard [12]. It is an invasive procedure done under general anesthesia and has some morbidity [7]. Some authors suggested that flexible bronchoscopy is a useful procedure for foreign body retrieval from infants and children with a 91.3% success rate [13]. Rigid or flexible bronchoscopy especially in pediatric patients remains a controversy and depends on the institute protocol. In our study all patients underwent rigid bronchoscopy initially. In 4 cases where incomplete retrieval was done, flexible bronchoscopy was used for inspection followed by a rigid bronchoscopy for retrieval.

In our study nature of foreign body aspirated were mostly organic 81.1% and inorganic foreign body accounted for 18.9%. Routinely found FB was peanut being present in 43.24% patients.

Many authors have reported the disparity between aspiration and hospital admission was more than 24 h and even after that there was a delay in diagnosis [14]. Some of the larger reviews have documented delays ranging from 20 to 40% of the patients reviewed [15]. In this present study, 54.04% of the patients reviewed had delayed presentation.

Delay in presentation were due to unintentional aspiration by child unnoticed by parents, vague clinical history, lack of respiratory symptoms, the unwillingness of the treating physician for getting a chest X ray and late referral. The severity of symptoms depends on the degree of obstruction of the tracheobronchial tree so it can be complete or incomplete and site of obstruction. If there is complete obstruction these patients are referred early. Delay usually occurs in patients with incomplete obstruction with normal chest X ray. A lack of history of inhalation most commonly led to delayed presentation and resulted in various respiratory complications like pneumonia, obstructive emphysema, atelectasis, pneumothorax and pneumomediastinum [16, 17]. Literature reveals there is a 7% incidence of pre hospital deaths in cases of foreign body aspiration [16].

Another factor that causes preoperative complications associated with FBA is the lack of standard treatment guidelines and management procedures [6].

Based on our study, we have developed an algorithm for management on the basis of outcomes.

## Conclusion

Experience from our institute suggests that the majority of complication occurs only when there is a major airway obstruction. Early diagnosis and referral reduces complication and mortality. Initial chest ray is a useful tool and in a crunch situation never be indecisive for doing a rigid bronchoscopy. Prophecy of negative bronchoscopy will probably counterbalance when it gets to saving the child.

## Data Availability

The datasets used and/or analyzed during the current study are available from the corresponding author on reasonable request.

## Abbreviations

FBA: Foreign body aspiration
FB: Foreign body

## References

[1] Janahi IA, Khan S, Chandra P, Al-Marri N, Saadoon A, Al-Naimi L (2017). A new clinical algorithm scoring for management of suspected foreign body aspiration in children. BMC Pulm Med.

[2] National Center for Injury Prevention and Control.Web-based Injury Statistics Query and Reporting System (WISQARS). Atlanta: CDC; 1999-2001. https://www.cdc.gov/injury/wisqars/index.html.

[3] Foltran F, Ballali S, Rodriguez H, van As AB, Passali D, Gulati A (2013). Inhaled foreign bodies in children: a global perspective on their epidemiological, clinical, and preventive aspects. Pediatr Pulmonol.

[4] Lifschultz BD, Donoghue ER (1996). Deaths due to foreign body aspiration in children: the continuing hazard of toy balloons. J Forensic Sci.

[5] Hanba C, Cox S, Bobian M, Svider PF, Gonik NJ, Shkoukani MA (2017). Consumer product ingestion and aspiration in children: a 15-year review. Laryngoscope.

[6] Yang XJ, Zhang J, Chu P, Guo YL, Tai J, Zhang YM (2016). Pneumomediastinum secondary to foreign body aspiration: clinical features and treatment explorement in 39 pediatric patients. Chin Med J.

[7] Zerella JT, Dimler M, McGill LC, Pippus KJ (1998). Foreign body aspiration in children: value of radiography and complications of bronchoscopy. J Pediatr Surg.

[8] Metrangolo S, Monetti C, Meneghini L, Zadra N, Giusti F (1999). Eight years’ experience with foreign-body aspiration in children: what is really important for a timely diagnosis?. J Pediatr Surg.

[9] Haliloglu M, Ciftci AO, Oto A, Gumus B, Tanyel FC, Senocak ME (2003). CT virtual bronchoscopy in the evaluation of children with suspected foreign body aspiration. Eur J Radiol.

[10] Hong SJ, Goo HW, Roh JL (2008). Utility of spiral and cine CT scans in pediatric patients suspected of aspirating radiolucent foreign bodies. Otolaryngol Head Neck Surg.

[11] Kosucu P, Ahmetoglu A, Koramaz I, Orhan F, Özdemir O, Dinç H (2004). Low-dose MDCT and virtual bronchoscopy in pediatric patients with foreign body aspiration. Am J Roentgenol.

[12] Adaletli I, Kurugoglu S, Ulus S, Ozer H, Elicevik M, Kantarci F (2007). Utilization of low-dose multidetector CT and virtual bronchoscopy in children with suspected foreign body aspiration. Pediatr Radiol.

[13] Ramírez-Figueroa JL, Gochicoa-Rangel LG, Ramírez-San Juan DH, Vargas MH (2005). Foreign body removal by flexible fiberoptic bronchoscopy in infants and children. Pediatr Pulmonol.

[14] Losek JD (1990). Diagnostic difficulties of foreign body aspiration in children. Am J Emerg Med.

[15] Kim IG, Brummitt WM, Humphry A, Siomra SW, Wallace WB (1973). Foreign body in the airway: a review of 202 cases. Laryngoscope.

[16] Hidaka H, Obara T, Kuriyama S, Kurosawa S, Katori Y, Kobayashi T (2013). Logistic regression analysis of risk factors for prolonged pulmonary recovery in children from aspirated foreign body. Int J Pediatr Otorhinolaryngol.

[17] Ciftci AO, Bingöl-Koloğlu M, Şenocak ME, Tanyel FC, Büyükpamukçu N (2003). Bronchoscopy for evaluation of foreign body aspiration in children. J Pediatr Surg.

